# Static beam tomotherapy as an optimisation method in whole‐breast radiation therapy (WBRT)

**DOI:** 10.1002/jmrs.232

**Published:** 2017-06-04

**Authors:** Matthew Squires, Yunfei Hu, Mikel Byrne, Ben Archibald‐Heeren, Sonja Cheers, Bruno Bosco, Amy Teh, Andrew Fong

**Affiliations:** ^1^ Precision Cancer Care Australia; ^2^ Radiation Oncology Centres Gosford New South Wales Australia; ^3^ Radiation Oncology Centres Wahroonga New South Wales Australia

**Keywords:** Breast cancer, dosimetry, radiation therapy, TomoDirect, tomotherapy

## Abstract

**Introduction:**

TomoTherapy (Accuray, Sunnyvale, CA) has recently introduced a static form of tomotherapy: *TomoDirect*™ (TD). This study aimed to evaluate TD against a contemporary intensity modulated radiation therapy (IMRT) alternative through comparison of target and organ at risk (OAR) doses in breast cancer cases. A secondary objective was to evaluate planning efficiency by measuring optimisation times.

**Methods:**

Treatment plans of 27 whole‐breast radiation therapy (WBRT) patients optimised with a tangential hybrid IMRT technique were replanned using TD. Parameters included a dynamic field width of 2.5 cm, a pitch of 0.251 and a modulation factor of 2.000; 50 Gy in 25 fractions was prescribed and planning time recorded. The planning metrics used in analysis were ICRU based, with the mean PTV minimum (D_99_) used as the point of comparison.

**Results:**

Both modalities met ICRU50 target heterogeneity objectives (TD D_99_ = 48.0 Gy vs. IMRT = 48.1 Gy, *P* = 0.26; TD D_1_ = 53.5 Gy vs. IMRT = 53.0 Gy, *P* = 0.02; Homogeneity index TD = 0.11 vs. IMRT = 0.10, *P* = 0.03), with TD plans generating higher median doses (TD D_50_ = 51.1 Gy vs. IMRT = 50.9 Gy, *P* = 0.03). No significant difference was found in prescription dose coverage (TD V_50_ = 85.5% vs. IMRT = 82.0%, *P* = 0.09). TD plans produced a statistically significant reduction in V_5_ ipsilateral lung doses (TD V_5_ = 23.2% vs. IMRT = 27.2%, *P* = 0.04), while other queried OARs remained comparable (TD ipsilateral lung V_20_ = 13.2% vs. IMRT = 14.6%, *P* = 0.30; TD heart V_5_ = 2.7% vs. IMRT = 2.8%, *P* = 0.47; TD heart V_10_ = 1.7% vs. IMRT = 1.8%, *P* = 0.44). TD reduced planning time considerably (TD = 9.8 m vs. IMRT = 27.6 m, *P* < 0.01), saving an average planning time of 17.8 min per patient.

**Conclusions:**

TD represents a suitable WBRT treatment approach both in terms of plan quality metrics and planning efficiency.

## Introduction

Breast conservation surgery followed by whole‐breast radiation therapy (WBRT) is a widely accepted treatment approach for patients presenting with early‐stage breast cancer.^1^, ^2^ Recent technical advances have resulted in a transition away from conventional tangential approaches and towards more complex methodologies, such as inverse planned intensity modulated radiation therapy (IP‐IMRT) and helical techniques.^3^, ^4^ These advances have sought to enhance normal tissue sparing and improve target homogeneity, as well as provide a platform for a simultaneous integrated boost to the tumour bed.^5^, ^6^

TomoTherapy (Accuray, Sunnyvale, CA) has recently introduced topotherapy,^7^ a static form of tomotherapy, to increase the versatility of its helical system. Branded *TomoDirect*™ (TD), the technology enables fixed beam treatments by moving the patient through the machine bore while maintaining specified beam angles. The platform complements the tomotherapy system which combines megavoltage computed tomography (MVCT) image guidance with a single‐energy 6‐MV intensity modulated fan beam. Image registration adjustments are applied to both the couch (translation) and gantry (roll) to facilitate precision in treatment delivery.

Non‐helical static beam techniques such as IMRT and TD are well suited to WBRT. These treatment modes avoid the low‐dose integral splay and long treatment times associated with helical approaches by confining dose delivery to tangential angles.^8^ The primary concern of low‐dose splay is the potential risk of secondary malignancy, particularly in the contralateral breast.^9^ This risk is accentuated in younger patients with early‐stage breast cancer, where cure rates are high and life expectancy is substantial.^9^ Static beam angle approaches aim to maximise the therapeutic ratio by ensuring that the tumour control probability (TCP) significantly outweighs the associated normal tissue complication probability (NTCP).^10^, ^11^, ^12^

As deliverable modulation has advanced, so too has the ability to homogenise dose. Several authors have demonstrated the benefits of improved target homogeneity, such as a reduction in oedema, breast pain and improved breast cosmesis.^13^, ^14^, ^15^ IP‐IMRT and TD offer the potential of optimal target homogeneity while limiting dose to normal tissue and critical structures.^4^, ^16^, ^17^ This study aimed to evaluate TD against a contemporary IP‐IMRT alternative through comparison of target and organ at risk (OAR) doses. A secondary objective was to evaluate planning efficiency by measuring optimisation times.

## Methods

Independent ethics approval was granted by the Oncology Research Australia (ORA) research committee.

Reports from the International Commission on Radiation Units and Measurements (ICRU) 50, 62 and 83 are designed to implement consistency and enable reliable interdepartmental reporting and analysis.^18^, ^19^, ^20^ Planning metrics used in analysis were based around these ICRU guidelines. The mean PTV minimum (D_99_) was chosen as the point of comparison. Statistical analysis was done using Student's unpaired single‐tailed *t*‐test; *P* < 0.05 was considered statistically significant.

### Patient selection

Twenty‐seven women treated during 2015 using IP‐IMRT WBRT were randomly selected from across two clinics: Radiation Oncology Centres, Gosford (*n* = 21), and Radiation Oncology Centres, Wahroonga (*n* = 6), and replanned using TD. All patients were simulated using customised vacbag immobilisation (Bionix, Toledo, OH) with both arms positioned overhead. Static free breathing scans were acquired for each patient: Gosford images with a GE kV CT scanner (GE Healthcare, Little Chalfont, United Kingdom) using 2.5‐mm slice intervals and Wahroonga images with a Somotom Definition Flash (Siemens Healthcare, Erlangen, Germany) using a 2‐mm slice thickness. The sample consisted of 6 left‐sided and 21 right‐sided WBRT cases.

### Regions of interest

For each patient, a planning target volume (PTV) defining the whole visible breast was delineated by a radiation oncologist. The PTV was derived using cues from external radiopaque markers placed by radiation therapists at simulation as well as discernible breast tissue evident on CT images. The PTV was limited by the external contour minus 3 mm and the ipsilateral lung and heart plus 4 mm. Efforts were made to ensure PTV consistency practice wide.

**Figure 1:**
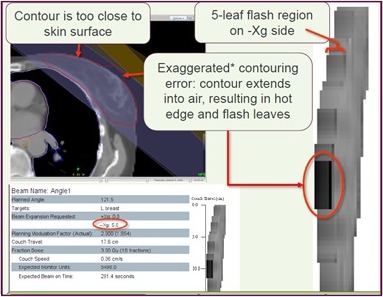
Areas of increased superficial fluency are depicted by darker areas on the tomotherapy treatment planning system.^22^ Such undesirable dosimetry is avoided by using a retracted planning target volume as an optimisation structure.

The retracted PTV enabled it to function as a planning optimisation structure. When optimising to superficial volumes, optimisation functions will try to compensate for the lack of build‐up and lateral scatter on the surface by using high superficial photon fluences, particularly when exclusively tangential beams are used.^21^ This may result in unnecessarily high superficial monitor unit (MU) delivery, causing high skin doses.^21^ Retracting the PTV from the surface avoids this potential compromise in plan quality, as shown in Figure 1.

To account for intrafraction motion as well as random and systematic errors, the field aperture was widened to overshoot the PTV anteriorly by at least 2 cm. Radiation therapists added relevant OARs including the ipsilateral lung and heart. The heart OAR was confined to the pericardial cavity and excluded superiorly located major vessels, as per Radiation Therapy Oncology Group (RTOG) guidelines.^23^ A CTV was subsequently added around the lumpectomy site by a radiation oncologist.

### IMRT technique

**Figure 2:**
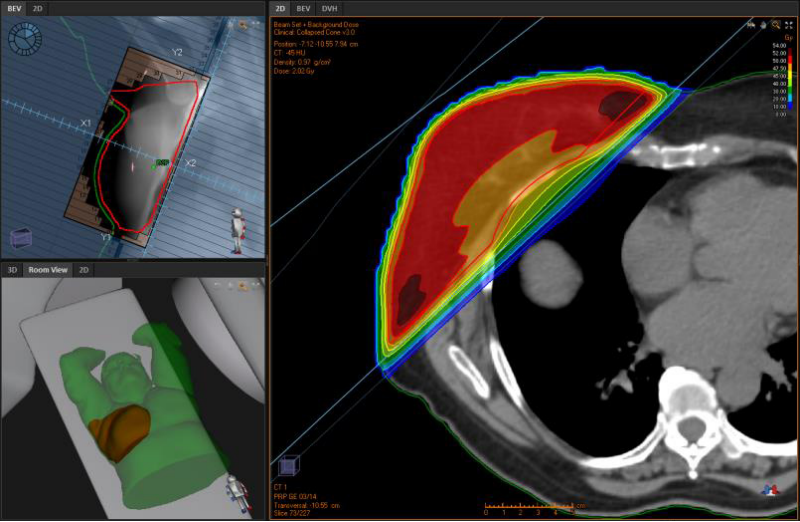
Intensity modulated radiation therapy technique: typical setup and dosimetry. [RayStation v4.5 (Raysearch, Stockholm, Sweden)].

The IMRT planning technique consisted of two tangential open parallel opposed beams and two tangential IP‐IMRT parallel opposed beams utilising the same gantry angles. The plans were prepared in RayStation v4.5 (Raysearch, Stockholm, Sweden) for a conventional linear accelerator capable of delivering both 6‐ and 10‐MV photon beams. The isocentre was placed on the posterior edge of the beams to minimise integral penumbra and conformal beam angles chosen to reduce dose to normal tissue. The open beams delivered a minimum of 60% of the prescription dose to the PTV, with up to 95% delivered anteriorly due to the breast contour. The open beams maintained approximately 2 cm of flash to ensure adequate dose coverage in the event of target deformation and intrafraction motion. The energy chosen was either 6 or 10 MV, guided in part by patient separation (6 MV *n* = 12; 10 MV *n* = 15). The IP‐IMRT beams acted as an optimised wedge to preferentially increase the dose coverage of the PTV edge bordering the ipsilateral lung. IP‐IMRT beams consisted of 6‐MV static ‘step and shoot’ multi‐leaf collimators (MLCs) (Gosford, *n* = 21) or 6‐MV dynamic MLCs (Wahroonga, *n* = 6); 50 Gy in 25 fractions was prescribed with the essential goal of PTV D_99_ > 47.5 Gy. Optimisation criteria were added with the aim of maximising target homogeneity and conformity while minimising OAR dose. The typical setup and dosimetry are depicted in Figure 2.

### TD technique

**Figure 3:**
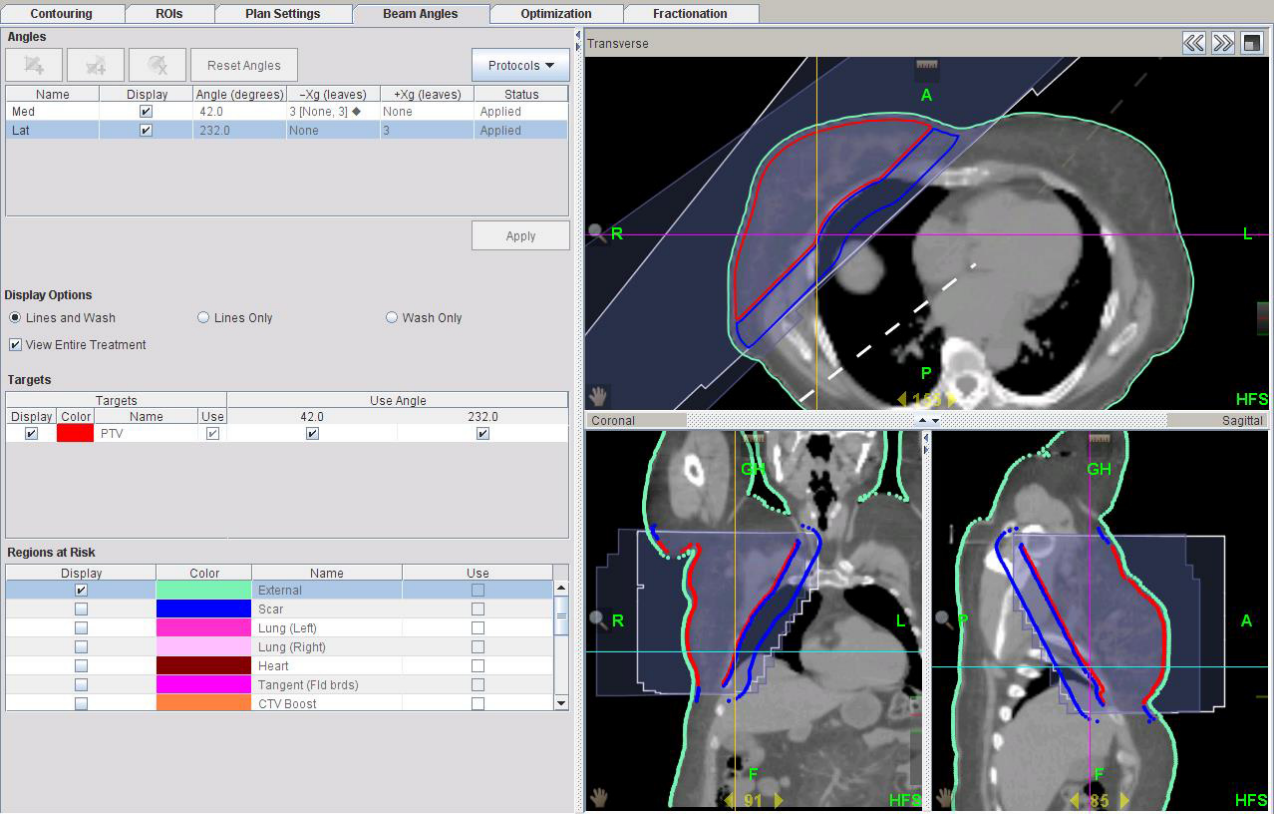
TomoDirect (TD) technique. No organs at risk were included in optimisation, besides a dose control volume of +0.2 cm to +2.0 cm placed along the posterior edge of the planning target volume. At least 2 cm of optimised flash was included to ensure adequate coverage in the case of target deformation.

Plans previously optimised with the IMRT technique (*n* = 27) were replanned using TomoTherapy's topotherapy platform: *TomoDirect*™ (TD) (Fig. 3). Identical medial and lateral beam incident angles were utilised to maintain setup consistency, with the only available (6 MV) beam energy utilised. TD parameters included a dynamic field width (FW) of 2.5 cm, a (default) pitch of 0.251 and a modulation factor (MF) of 2.000.

The FW is the collimator defined field size in the longitudinal direction; it can be fixed to one of three settings: 1, 2.5 or 5 cm (defined at the isocentre). Varying the FW will affect the sharpness of dose fall‐off in the longitudinal direction, as well as the modulation possible over the target. A smaller FW will increase treatment delivery times; a 2.5‐cm FW was chosen to achieve a balance between satisfactory treatment time and suitable modulation. The selection of dynamic jaws, or *TomoEdge*™, allowed the FW to reduce to 1 cm directly before and after the target. This recent hardware upgrade allowed for a reduction in longitudinal penumbra without a significant compromise to delivery time. The MF is an indicator of the degree of intensity modulation permitted, typically ranging from 1.4 to 3.0. A high MF can affect both treatment and planning time, since a higher degree of modulation will require more iterations to optimise. The chosen modulation factor of 2.000 reflected typical practice wide modulation. In TD mode, the pitch is the direct outcome of the FW; it is defined as the distance of couch travel in centimetres per sinogram projection.^11^

No OARs were included in optimisation, besides a dose control volume (DCR) of +0.2 cm to +2.0 cm placed along the posterior edge of the PTV. At least 2 cm of flash was included to ensure adequate dose coverage in the case of target deformation. Because TD fields are characterised by heterogeneous fluence, adding flash involved projecting the average fluence of the two closest leaves intersecting the PTV. This offered a significant dosimetric advantage over IMRT:^24^ the IMRT hybrid technique only provided flash from the open beam portion; TD flash provided optimised flash for the entire beamset.

**Figure 4:**
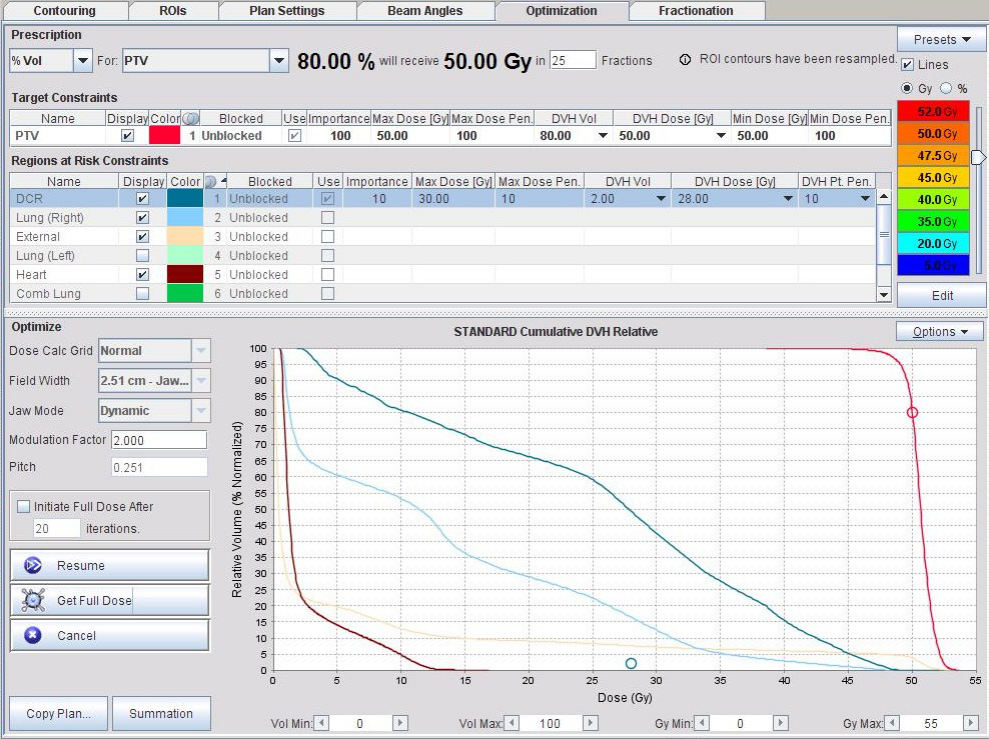
Typical target optimisation parameters included a goal of 80% of the planning target volume receiving 50 Gy. The dose control volume allowed integral dose limitation and was routinely set to 60% of the prescribed dose (30 Gy), with D_2_ set to 28 Gy.

Typical target optimisation parameters (Fig. 4) included the goal of 80% of the PTV receiving 50 Gy to ensure that the PTV D_99_ exceeded 47.5 Gy. This starting point enabled quick iterative progress towards achieving the ICRU50 heterogeneity limits of +7% (53.5 Gy) and −5% (47.5 Gy).^18^ The DCR allowed integral dose limitation and was routinely set to 60% of the prescribed dose (30 Gy), with D_2_ set to 28 Gy.

### QA

TD plans underwent individual quality assurance (QA) using ArcCHECK (Sun Nuclear, Melbourne, FL), with a gamma tolerance of 3 mm and 3% and a threshold of 10%. Plans with less than 95% of points passing the gamma analysis were rejected and replanned to ensure the accuracy of treatment delivery. IMRT plans had their open beams verified using RadCalc (LifeLine Software, Austin, TX), with up to 5% calculated deviation deemed acceptable. IP‐IMRT beams were verified using ArcCHECK (Sun Nuclear, FL), with a gamma tolerance of 3 mm and 3% and a threshold of 10%, using the same pass criterion as TD plans.

## Results

### PTV metrics

There was no significant difference found in the PTV D_99_ > 47.5 Gy point of comparison (TD D_99_ = 48.0 Gy vs. IMRT = 48.1 Gy, *P* = 0.26). Prescription dose (50 Gy) coverage was also statistically similar (TD V_50_ = 85.5% vs. IMRT = 82.0%, *P* = 0.09) (Table 1).

**Table 1:** Dosimetric comparisons: planning target volume (PTV)

Characteristic	TomoDirect		Inverse planned IMRT		*P*‐value
D_99_	48.0 Gy	(0.47)	48.1 Gy	(0.58)	0.26
D_50_	51.1 Gy	(0.45)	50.9 Gy	(0.38)	0.03^a^
D_1_	53.5 Gy	(1.11)	53.0 Gy	(0.35)	0.02^a^
V_50_	85.5%	(7.43)	82.0%	(11.59)	0.09
HI	0.110	(0.029)	0.099	(0.015)	0.03^a^

(Standard deviation; σ):

D_99_ is the dose received by 99% of the PTV – near minimum.

D_50_ is the dose received by 50% of the PTV – median.

D_1_ is the dose received by 1% of the PTV – near maximum.

V_50_ is the volume of PTV receiving the prescribed dose of 50 Gy.

HI, homogeneity index, calculated as (D_1_–D_99_)/50.

a Statistically significant difference, defined as *P* < 0.05.

PTV maximum doses were defined as the dose received by 1% of the PTV (D_1_). This reporting approach rendered higher near maximum doses than the D_2_ criterion of ICRU83,^20^ but was consistent with shared departmental protocols. The mean D_1_ for the TD arm was 53.5 Gy compared to 53.0 Gy for the IMRT subset (*P* = 0.02). Variation was higher within the TD group (σ: 1.11 vs. 0.35), a phenomenon most likely due to the effect of patient separation and the unavailability of higher TD beam energies. TD plans were also found to have generated higher median doses (TD = 51.1 Gy, σ = 0.45 vs. IMRT = 50.9 Gy, σ = 0.38, *P* = 0.03). Nevertheless, the mean of both groups met ICRU50 target heterogeneity goals (+7% to −5%).^18^

**Figure 5:**
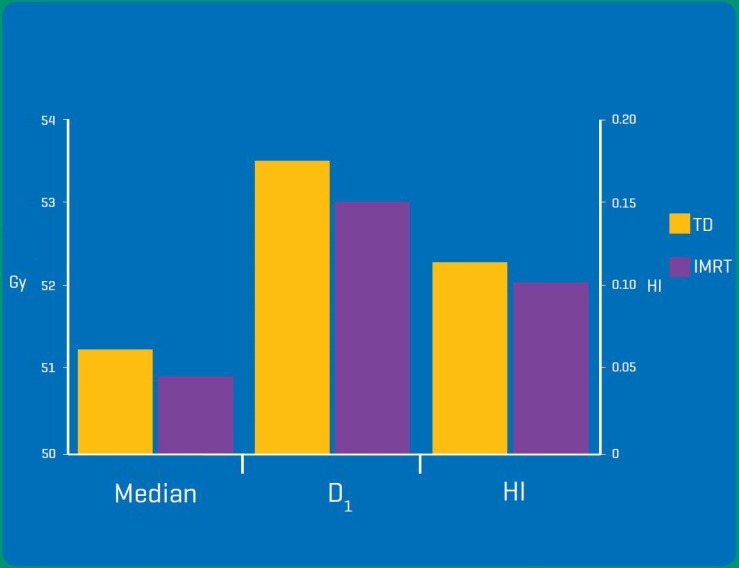
Statistically significant differences (*P* < 0.05) were found in the median (dose received by 50% of the planning target volume (PTV)), D_1_ (dose received by 1% of the PTV) and HI (homogeneity index) of the PTV.

IMRT plans demonstrated a statistically significant advantage in target homogeneity (HI TD = 0.11 vs. IMRT = 0.10, *P* = 0.03). While there are various HI formulae applied within literature, ICRU83 advocates the definition (D_2_–D_98_)/D_50_.^20^ We modified this formula slightly, defining HI as (D_1_–D_99_)/50 Gy, primarily to maintain internal consistency given the utilisation of D_99_ and D_1_ data. Figure 5 illustrates the statistically significant differences of target metrics.

### OAR metrics

TD plans produced a statistically significant reduction in V_5_ ipsilateral lung doses (TD V_5_ = 23.2% vs. IMRT = 27.2%, *P* = 0.04), of which clinical significance is uncertain. Other queried OAR metrics remained comparable (TD ipsilateral lung V_20_ = 13.2% vs. IMRT = 14.6%, *P* = 0.30; TD heart V_5_ = 2.7% vs. IMRT = 2.8%, *P* = 0.47; TD heart V_10_ = 1.7% vs. IMRT = 1.8%, *P* = 0.44) (Table 2).

**Table 2:** Dosimetric comparisons: organs at risk

Characteristic	TomoDirect		Inverse planned IMRT		*P*‐value
Heart V_5_	2.7	(4.65)	2.8	(5.16)	0.47
Heart V_10_	1.7	(3.47)	1.8	(3.62)	0.44
Ipsilateral lung V_5_	23.2	(7.28)	27.2	(9.02)	0.04^a^
Ipsilateral lung V_20_	13.2	(7.15)	14.6	(6.49)	0.30

(Standard deviation; σ):

Heart V_5_: Volume of heart receiving 5 Gy (%).

Heart V_10_: Volume of heart receiving 10 Gy (%).

Ipsilateral lung V_5_: Volume of ipsilateral lung receiving 5 Gy (%).

Ipsilateral lung V_20_: Volume of ipsilateral lung receiving 20 Gy (%).

a Statistically significant difference, defined as *P* < 0.05.

### Optimisation time

**Figure 6:**
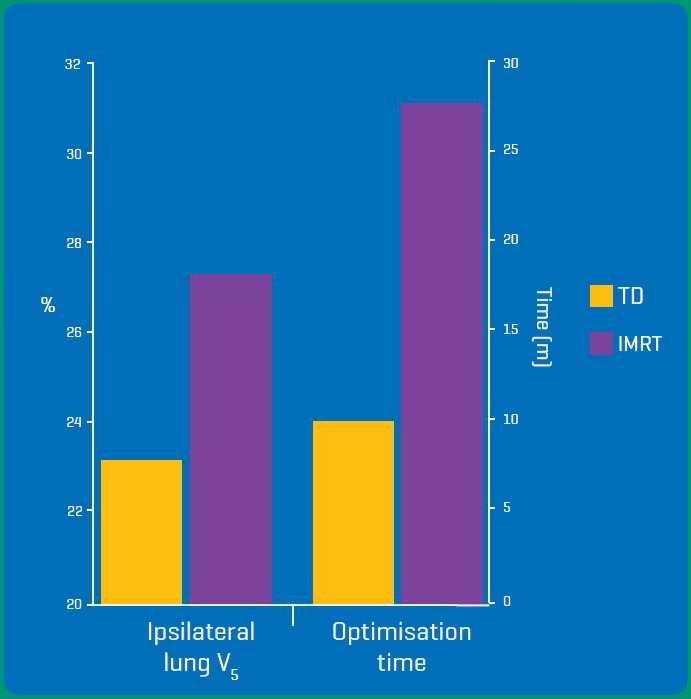
Statistically significant differences (*P* < 0.05) were found between the ipsilateral lung (V_5_) doses and optimisation times.

The time required to reach sufficient dose optimisation was recorded in both study arms (Fig. 6). TD was found to reduce planning time considerably (TD = 9.8 m vs. IMRT = 27.6 m, *P* < 0.01) and reduce variation in planning time (TD σ = 2.77 vs. IMRT = 4.33). The considerable mean time saved per case (17.8 min) was compounded by the ease of data transfer from the TomoTherapy treatment planning system to the patient record and verify system.

## Discussion

### The energy effect

Tomotherapy is limited to a single 6‐MV treatment energy. The lack of a higher energy option restricted the ability to lessen maximum (D_1_) doses, particularly in patients with large PTV posterior edge (PE) separations. This limitation was reported by Fields et al., among others, who found that PE separations >21 cm often produced hotspots >110%.^4^, ^25^, ^26^, ^27^, ^28^ Similarly Das et al.^26^ found a correlation between hotspots >115% and chest wall separations >22 cm.

**Figure 7:**
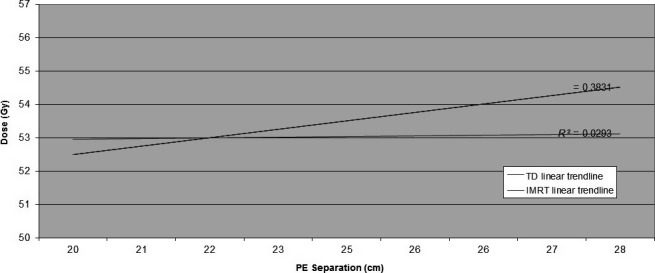
Linear regression analysis: maximum (D_1_) dose versus planning target volume posterior edge (PE) separation. Intensity modulated radiation therapy plans generated lower D_1_ doses once PE exceeded 22.0 cm (TD *R* ^2^ = 0.3831; IMRT = 0.0293).

A linear regression analysis of our own data revealed the extent to which higher energies (10 MV) had the ability to reduce maximum (D_1_) PTV doses. Once PE separation reached 22 cm, the differential advantage of the 10‐MV option available in IMRT plans became evident, as demonstrated in Figure 7.

This data may be useful to screen patients who are potentially better suited to higher energy IMRT at the time of simulation.

### Static beam angle versus helical techniques

The TomoTherapy HD platform allows for helical delivery over a rotational interval of 7°, up to a total of 51 projections for each gantry revolution. With excellent high‐dose conformity, Haciislamoglu et al.^3^ among others argue the merits of helical delivery in WBRT. Franco et al.^11^ state the obvious drawback of helical techniques: larger integral dose. This low‐dose bath has been shown to be the genesis of secondary malignancy, particularly in the contralateral breast.^9^ Static beam angles facilitate avoidance of tissues outside of the treatment path, with healthy tissues receiving only minor scatter radiation. This was demonstrated by our results, particularly in the low V_5_ ipsilateral lung doses (TD V_5_ = 23.2%; IMRT = 27.2%). We suspect this was partly due to the use of dynamic jaws, or *TomoEdge*™, which constrains longitudinal penumbra. Static beam angles are the safest approach when seeking to reduce risk in younger breast cancer patients, who potentially benefit only minimally from WBRT.^11^

A recognised benefit of static beam techniques is the reduction in treatment delivery time.^11^ Helical tomotherapy may employ the use of complete and directional ‘blocks’ to preclude radiation from specific volumes. These blocks result in prolonged treatment times due to the constraint of irradiation from certain treatment angles while the gantry rotational velocity remains constant. Anecdotally TD and IMRT treatment delivery times are similar. Factors such as required collimator rotation, modulation and use of either static ‘step and shoot’ or dynamic IP‐IMRT options may impact treatment delivery time. The choice of a 5‐cm FW would likely offer a small time advantage to TD, with a modest compromise to modulation capacity.

TD imaging involves a MVCT helical approach, and we found that the time taken to acquire a typical 3‐mm reconstructed image was approximately 160 sec. This exceeded the conventional linear accelerator (kVCT) imaging time of approximately 30 sec (left sided 280–90°; right sided 182–345°), placing TD at a disadvantage in terms of its ability to image for deep inspiration breath hold (DIBH) procedures (patients are typically unable to maintain a breath hold for much longer than 30 sec).^29^

## Limitations

Inter‐user planning ability is a recognised uncontrolled variable in comparative plan analysis.^30^ The small sample size (*n* = 27) generated by multiple planners compounds the size of this human variable. Generation of TD plans was carried out by the same planner, while IMRT plans were the result of a mixture of radiation therapists of various planning proficiencies.

Given the likely efficiency impact, timing data were included. While optimisation times are also planner dependent, contouring time was excluded to reduce this variable. The magnitude of the time saved using TD (17.8 m or 65.5%; TD = 9.8 m vs. IMRT = 27.6 m, *P* < 0.01) would likely have an impact on tomotherapy cost economics. Tomotherapy capital and service costs should be weighed against potential workflow efficiency dividends.

The potential dosimetric enhancement offered by additional beam angles has been postulated by Fields et al.^25^ among others. Multiple beam angles attempt to give the dosimetrist control of the balance between homogeneity and increased integral dose, providing the ability to customise competing metrics to meet individual patient needs. The use of additional TD beam angles warrants further investigation to extend the scope and efficacy of the TD technique.

## Conclusions

TD represents a suitable WBRT alternative treatment approach. Despite producing plans with higher mean maximum doses, PTV metrics maintained adherence to ICRU guidelines. Optimised flash allowed for confidence in adequate dose coverage in the case of target deformation. OAR metrics were comparable, with TD demonstrating a lower ipsilateral lung dose. A principal TD advantage was found to be improved planning efficiency, with significantly lower optimisation times.

## Conflict of interest

The authors declare no conflict of interest.
